# Supplementary figures and images for: Formative mixed-method multicase study research to inform the development of a safer sex and healthy relationships intervention in further education (FE) settings: the SaFE Project
Source: BMJ Open. 2019 Jul 9;9(7):e024692. doi: 10.1136/bmjopen-2018-024692 (PMC6629453; doi:10.1136/bmjopen-2018-024692)

# Online Appendix 1: Safer sex and relationships in FE (SaFE): Intervention logic model

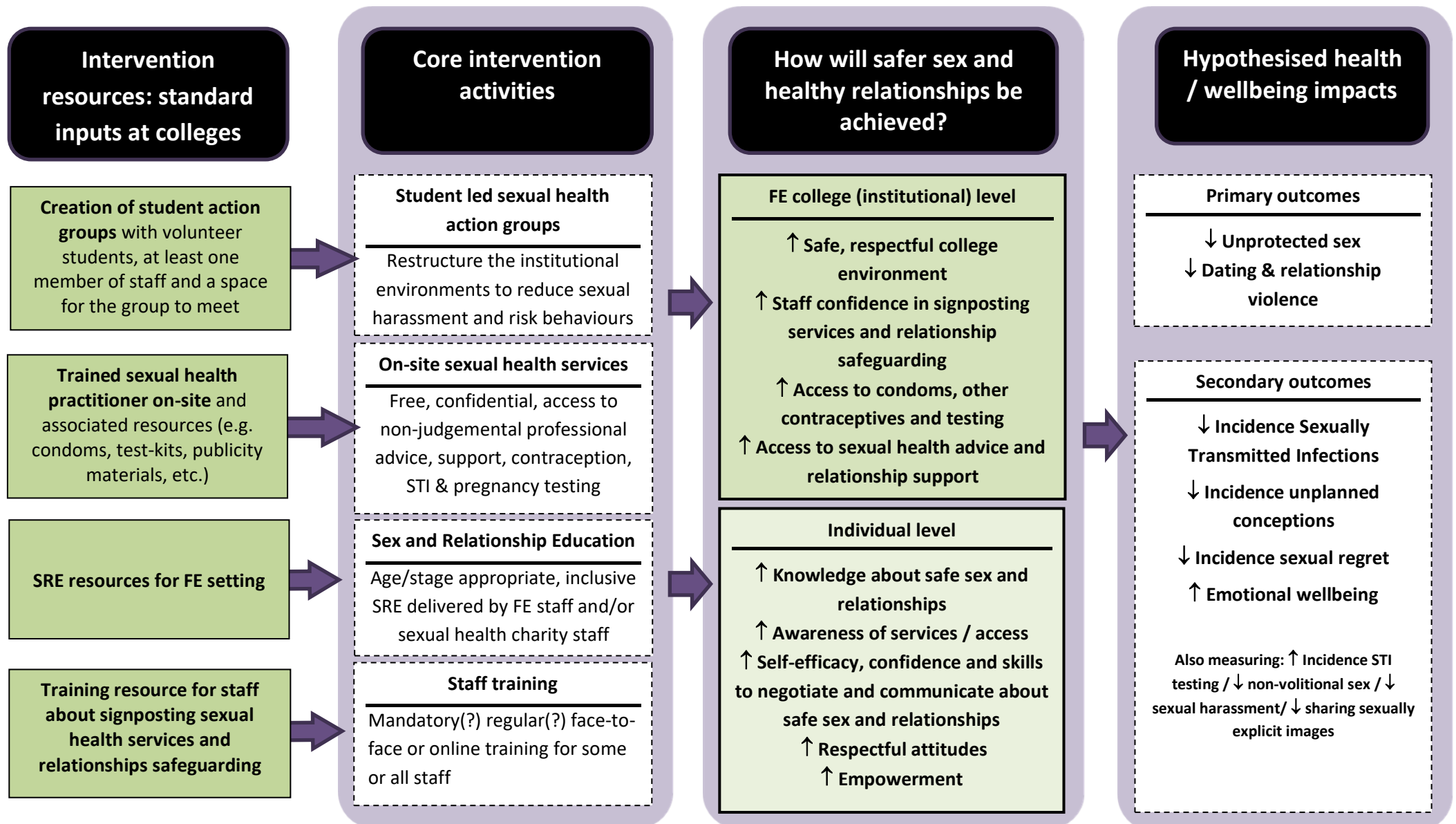

Supplement: Supplementary file 1 [file bmjopen-2018-024692supp001.pdf]
